# Large-scale gene expression changes in APP/PSEN1 and GFAP mutation models exhibit high congruence with Alzheimer’s disease

**DOI:** 10.1371/journal.pone.0291995

**Published:** 2024-01-18

**Authors:** Stephen C. Gammie, Albee Messing, Mason A. Hill, Cynthia A. Kelm-Nelson, Tracy L. Hagemann

**Affiliations:** 1 Department of Integrative Biology, University of Wisconsin-Madison, Madison, Wisconsin, United States of America; 2 Department of Comparative Biosciences, School of Veterinary Medicine, University of Wisconsin-Madison, Madison, Wisconsin, United States of America; 3 Waisman Center, University of Wisconsin-Madison, Madison, Wisconsin, United States of America; 4 Department of Surgery, Division of Otolaryngology-Head and Neck Surgery, University of Wisconsin-Madison, Madison, Wisconsin, United States of America; University of Florida, UNITED STATES

## Abstract

Alzheimer’s disease (AD) is a complex neurodegenerative disorder with both genetic and non-genetic causes. Animal research models are available for a multitude of diseases and conditions affecting the central nervous system (CNS), and large-scale CNS gene expression data exist for many of these. Although there are several models specifically for AD, each recapitulates different aspects of the human disease. In this study we evaluate over 500 animal models to identify those with CNS gene expression patterns matching human AD datasets. Approaches included a hypergeometric based scoring system that rewards congruent gene expression patterns but penalizes discordant gene expression patterns. The top two models identified were APP/PS1 transgenic mice expressing mutant APP and PSEN1, and mice carrying a GFAP mutation that is causative of Alexander disease, a primary disorder of astrocytes in the CNS. The APP/PS1 and GFAP models both matched over 500 genes moving in the same direction as in human AD, and both had elevated GFAP expression and were highly congruent with one another. Also scoring highly were the 5XFAD model (with five mutations in APP and PSEN1) and mice carrying CK-p25, APP, and MAPT mutations. Animals with the APOE3 and 4 mutations combined with traumatic brain injury ranked highly. Bulbectomized rats scored high, suggesting anosmia could be causative of AD-like gene expression. Other matching models included the SOD1G93A strain and knockouts for SNORD116 (Prader-Willi mutation), GRID2, INSM1, XBP1, and CSTB. Many top models demonstrated increased expression of GFAP, and results were similar across multiple human AD datasets. Heatmap and Uniform Manifold Approximation Plot results were consistent with hypergeometric ranking. Finally, some gene manipulation models, including for TYROBP and ATG7, were identified with reversed AD patterns, suggesting possible neuroprotective effects. This study provides insight for the pathobiology of AD and the potential utility of available animal models.

## Introduction

Alzheimer’s disease (AD) can lead to severe dementia and includes the formation of neurofibrillary tangles of tau proteins and beta-amyloid plaques as well as cortical atrophy [1]. AD is a complex, progressive disorder that is difficult to treat [2] and involves dysregulated expression of thousands of genes across multiple brain regions [3]. Animal models exist for various neurological disorders [4–6], including those for studying AD [7–10]. For many models, data on large-scale gene expression patterns in the central nervous system (CNS) exist as does data from human AD large-scale gene expression derived from postmortem tissue [3, 11–14]. Further, across multiple independent studies, common AD dysregulation patterns have been identified [15–17], suggesting that meaningful large-scale gene expression patterns exist with AD both within and across brain regions.

Multiple studies have compared animal models to human AD, including recent work that used large-scale gene expression from over 200 mouse models and identified significant matching to human AD-related co-expression modules from different brain regions [18]. The MAPT mutation, the CDK5-p25 model, and the 5XFAD model (that has five mutations in the APP and PSEN1 genes) matched multiple human gene co-expression modules. Further, models for other diseases, including amyotrophic lateral sclerosis (ALS), prion disease, and Huntington’s disease were also found to match some of the human AD modules. A separate study identified subtypes of AD gene expression in humans (A, B1, B2, C1, and C2) based on gene expression patterns (including co-expression modules) related to tau or amyloid-β pathology or other AD-related molecular pathways [19]. The C1 and C2 groups were found to be a robust match with the common amyloid-β predominant form of AD and had a high matching with 5XFAD and APP/PS1 models at the gene expression level [19].

Although neuronal dysfunction is often the focus of AD research, the glial neuroinflammatory response is a significant factor in disease pathology. Alexander disease (AxD) is a neurodegenerative disease caused by dominant gain-of-function mutations in the gene for GFAP (glial fibrillary acidic protein), an intermediate filament protein expressed by astrocytes in the CNS [20]. AxD-associated GFAP mutations lead to protein aggregation and reactive gliosis, with activation of stress response genes including GFAP itself [21]. We have previously proposed that as a primary disorder of astrocytes, AxD represents a model in which to study the specific effects of astrocyte dysfunction in neurodegenerative disease [22]. Here we provide new large-scale gene expression data from a mouse model of AxD [21, 23, 24] for comparison alongside AD and other animal models to assess the relevance of astrocyte pathology and function in AD.

In this study, we contribute to on-going work by comparing and ranking expression patterns from 500 animal models with those from human AD. We include the new data from the GFAP mutation mice along with two independently curated model lists; one produced by us (over 300 models) in a study of human depression [25] and the other from Wan and colleagues (~ 200 models) [18] in a study of AD. This study focusses on congruence (similarity) of dysregulation in the model and AD (same direction of change) that uses a standardized cutoff and is independent of whether a gene is in a co-expression module or not. For ranking models, we used a hypergeometric based scoring system that rewards common expression patterns (down or up in both model and human AD) but penalizes opposing expression patterns. Gene enrichment analysis was performed on overlapping genes. Multiple representations for human AD across and within regions were used, including a portrait we recently created using 22 human AD datasets from males and females across multiple CNS regions [15], separate male and female portraits, three other meta-analysis studies [16–18], 28 individual datasets from males and females in multiple brain regions, and subtypes of AD gene expression [19]. In our analysis we also use Rank Rank Hypergeometric Overlap (RRHO) heatmaps [26] and Uniform Manifold Approximation Plot (UMAP) [27] to compare models with human AD. As a final step, we also explore models that have opposite expression patterns as seen in AD as these could reflect gene pathways for reversing AD expression patterns. Together, the findings may provide new information on how AD gene expression patterns can emerge and how they may be reversed.

## Results

## Evaluation of models congruent with the AD portrait

**Fig 1:**
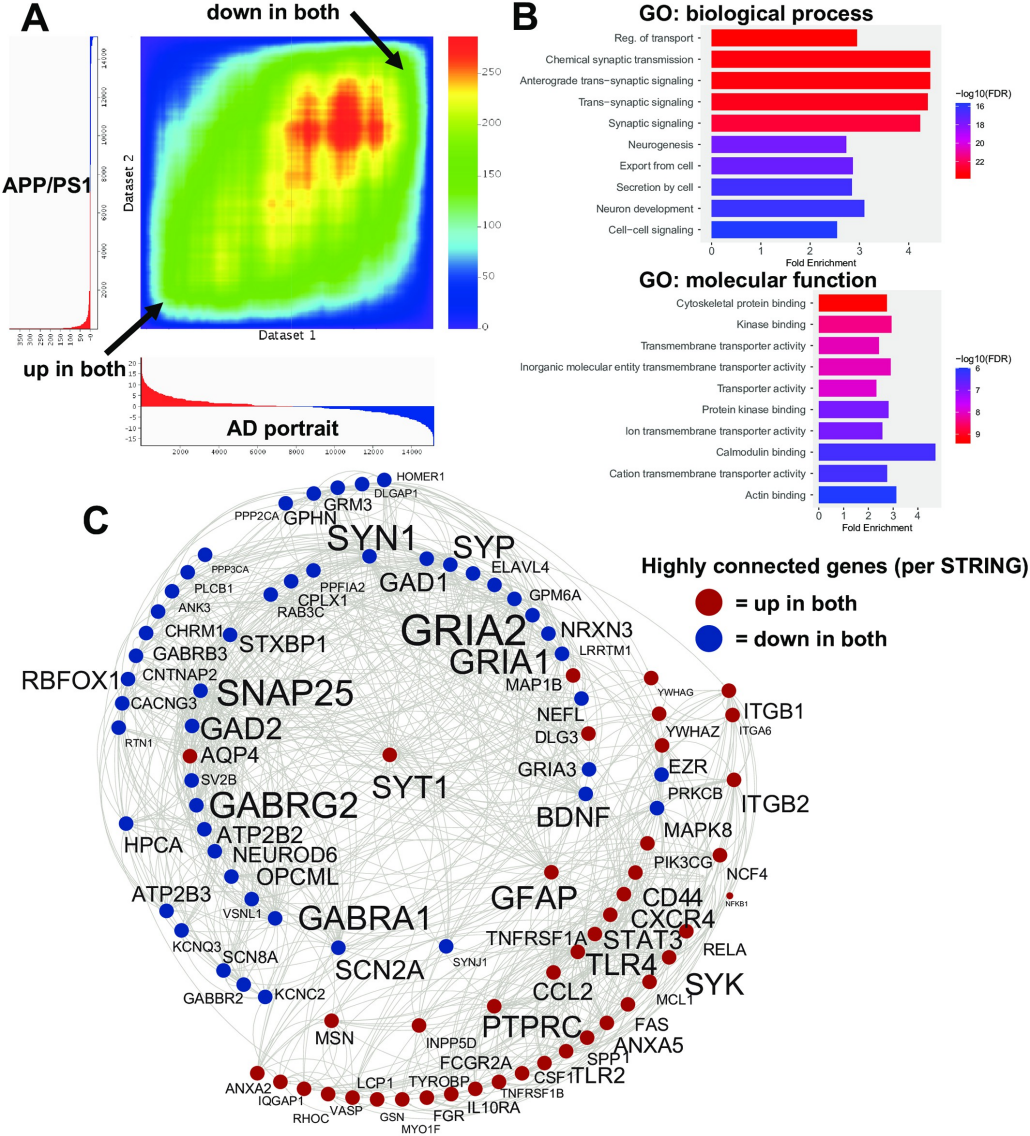
Congruent gene expression in APP/PS1 mice and human AD. A: An RRHO heatmap [26] of the AD portrait (X axis) with the APP/PS1 mice (top ranked model) (Y axis) indicates a high matching of up-up and down-down genes (arrows). Color is–log transformed hypergeometric p-value showing the strength of the overlap as positive or negative enrichment. In X and Y axes the profiles of upregulated genes are shown in red and downregulated genes in blue (some clipping of highly significant downregulated genes occurred in the model). B: Enrichment analysis of common up and down regulated genes is shown using ShinyGO [28]. C: Congruent genes with the highest levels of protein-protein interaction (determined via STRING [29]) are plotted in Cytoscape [30]. Interactions are highlighted by lines. AD and model upregulated genes are shown in red and AD and model downregulated genes are shown in blue. Increased size of font for gene symbol reflects higher number of connections between genes. Common genes of interest include: GFAP, BDNF, GRIA2, GRIA1, GABRA1, GABRG2, SNAP25, and PTPRC.

The hypergeometric scoring system rewarded same direction patterns, but also penalized opposite patterns and allowed for analysis of the 500+ models with human AD. Overall, compared with the AD portrait, the first, second, and third ranked model matches were for the APP/PSEN1 (APP/PS1) mouse model (known human gene mutations placed in mice) when examining hippocampus (10 months), frontal cortex from females (8 months), and frontal cortex from males (8 months), respectively (S1 File). For this model and many others, there were multiple datasets that represent different brain regions and sexes. For the top APP/PS1 match, 239 upregulated genes matched up genes in AD (out of the top 1000 up for both) and 276 downregulated genes matched genes down in AD. An RRHO heatmap of the relation of the top model with the AD portrait for all genes is shown in Fig 1A. Only 16 genes were in the opposite direction. The 515 congruent genes showed enrichment in a wide range of categories that relate to AD, including synaptic signaling, cell adhesion, neuron development, cytoskeleton binding, neurogenesis, wound healing, immune response, MAPK cascade, inflammatory response, and learning or memory (Fig 1B; S2 File). STRING analysis revealed a subset of highly connected genes/proteins that included GFAP, BDNF, GRIA1, GRIA2, SNAP25, GABRG2, GABRA1, SYN1, TLR4, and GAD2 (Fig 1C). In the AD portrait, the top dysregulated gene is ITPKB. This gene was upregulated in AD as well as in the APP/PS1 model. The top 20 animal models that are most congruent with the human AD portrait (where each model genotype is represented once) are shown in Table 1. The full ranking of all 500+ models is provided in S1 File.

**Table 1:** Top 20 models with highest congruence to human AD. Overall rank from over 500 models and ranking is based on the hypergeometric score that rewards matching in same direction (up-up and down-down) while penalizing opposite patterns. Full scoring details provided in S1 File. Each of the top models was from mice except for Flinders and bulbectomize which were from rats. Model numbers in lower case “m” are from our previous [25] and current study while upper case “M” with upper case “A” added are from a different study [18] with original numbering used. GEO Omnibus or Synapse numbers are provided. Abbreviations: E, embryonic day; KO, knockout; cKO, conditional KO; CP, caudate putamen; sCreutzfeld-Jakob, sporadic Creutzfeld-Jakob; and TBI, traumatic brain injury. References (if available) are shown under genotype.

rank	model #	GEO/syn #	genotype or treatment	sex	source; age
1	m328	GSE63943	APP/PS1 [10]		CA1 hippocampus; 10 months
4	m292	GSE197044	GFAP (R236H) (20)	male	hippocampus; day 56
6	M353		5XFAD [31]	both	hippocampus; 6 months
7	M19A	GSE102014	APOE3 with TBI [32]	female	cortex/hippocampus; 3 months
8	m133	GSE83336	Flinders [33]	male	ventral dentate gyrus
10	M14A	GSE100888	SOD1G93A [34]		spinal cord; day 150
13	M65A	GSE65159	CK-p25 model [35]	female	hippocampus; 3 months
14	M237	GSE977	GFAP overexpression [36]	both	olfactory bulb; 4 months
15	M24A	GSE27218	TDP-43 antisense [37]		striatum, 8–10 weeks
16	M22A	GSE102014	APOE4 with TBI [32]	male	cortex/hippocampus; 3 months
17	M243A	syn3157182	MAPT	male	forebrain; 4.5 months
22	M199A	GSE90977	sCreutzfeld-Jakob [38]		cortex; 180 days post infection
23	m83	GSE9789	bulbectomized [39]		frontoparietal cortex; 2 months
27	M206A	GSE98875	LSD1 [40]	female	hippocampus
28	m141	GSE139524	PWScrm+/- SNORD116 [41]		hypothalamus
31	m334	GSE60415	GNAS cKO [42]		medulloblastoma from cerebella
32	m102	GSE55314	GRID2 KO [43]	female	cerebellum; 12 weeks
38	m57	GSE46139	INSM1 KO [44]		pituitary; E17
39	m233	GSE11322	XBP1 KO [45]		telencephalon culture; E12.5
43	m258	GSE31458	MPTP treatment [46]		CP; three strains/transgenics

**Fig 2:**
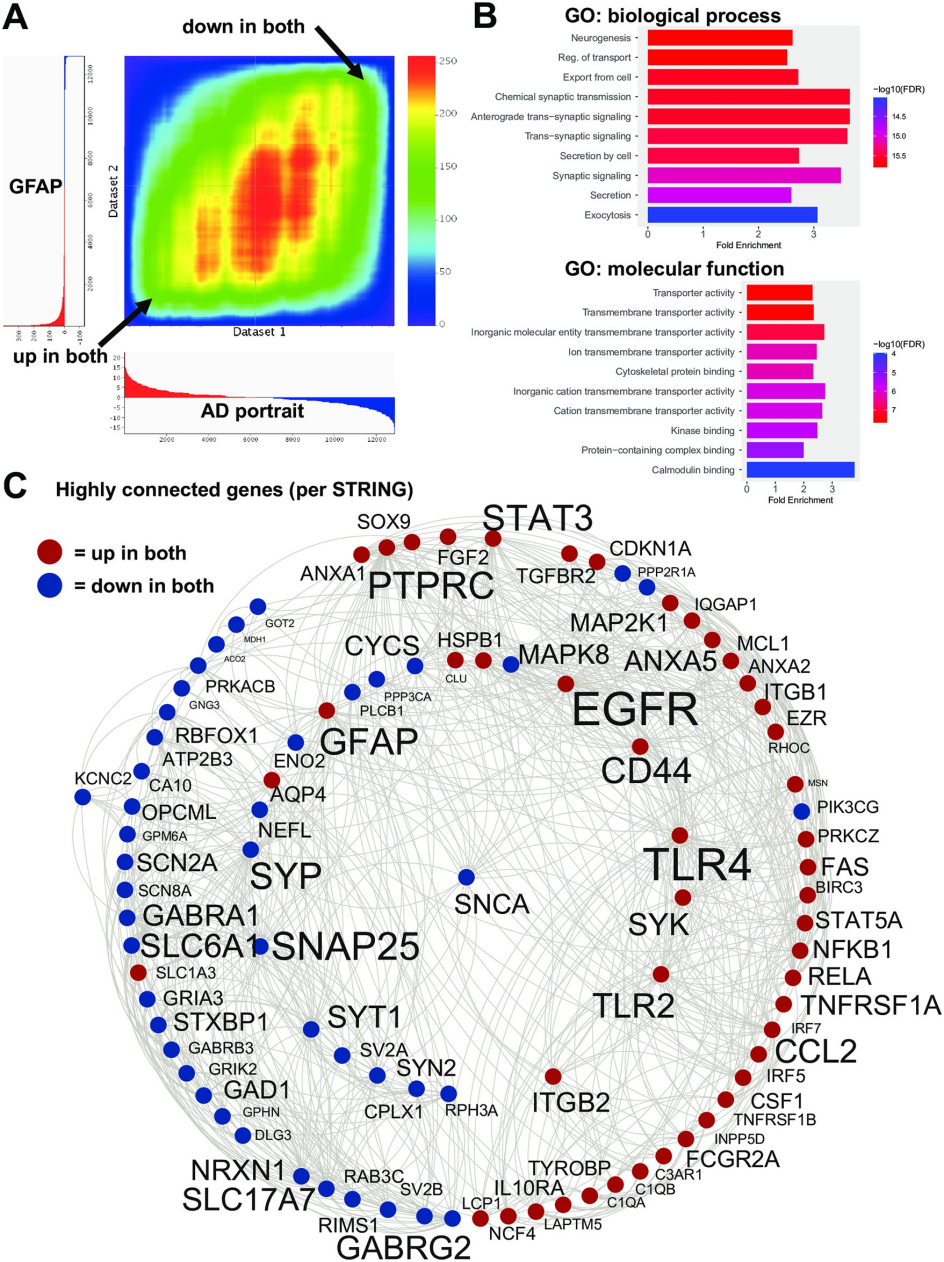
Congruent gene expression in GFAP mice and human AD. A: An RRHO heatmap [26] of the AD portrait (X axis) with the GFAP mutation mice (hippocampus; second ranked model) (Y axis) indicates a high matching of up-up and down-down genes (arrows). B: Enrichment analysis of common up and down regulated genes is shown using ShinyGO [28]. C: Congruent genes with the highest levels of protein-protein interaction (determined via STRING [29]) are plotted in Cytoscape [30]. Interactions are highlighted by lines. AD and model upregulated genes are shown in red and AD and model downregulated genes are shown in blue. Increased size of font for gene symbol reflects higher number of connections between genes. Common genes of interest include: GFAP, EGFR, TLR4, SNAP25, PTPRC, and SNCA.

The second best model and ranked fourth and fifth overall was an Alexander disease associated GFAP mutation (R236H) in adult mice at day 56 in hippocampus and corpus collosum, respectively. The GFAP mutation in young postnatal day 14 mice also ranked highly at 48^th^ (astrocytes in corpus collosum) and 62^nd^ (hippocampal astrocytes). A different Alexander disease model overexpressing wildtype human GFAP ranked 13^th^. For the top GFAP mutation match, 274 upregulated genes matched up genes in AD (out of the top 1000 up for both) and 271 downregulated genes matched genes down in AD. Only 33 genes were in the opposite direction. An RRHO heatmap of the relation of the top model with the AD portrait for all genes is shown in Fig 2A. The 545 congruent genes showed enrichment in a wide range of categories that relate to AD (Fig 2B, S2 File). STRING analysis revealed a subset of highly connected genes that included GFAP, EGFR, TLR4, SNAP25, PTPRC, SLC6A1, CD44, and SNCA (Fig 2C).

**Fig 3:**
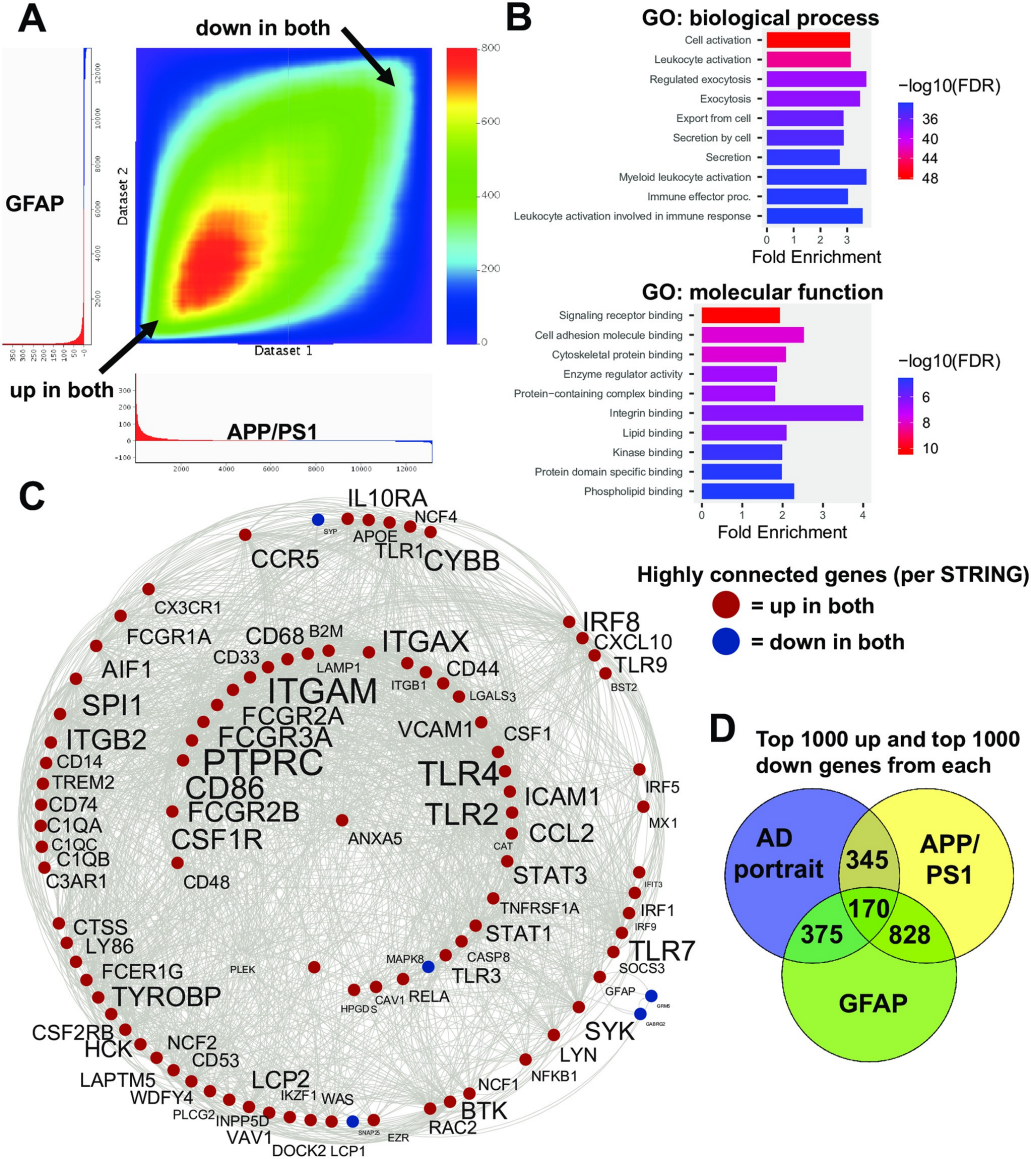
Congruent gene expression in APP/PS1 and GFAP mice. A: An RRHO heatmap [26] of the APP/PS1 (X axis) with the GFAP mutation mice (Y axis) indicates a high matching of up-up and down-down genes (arrows). B: Enrichment analysis of common up and down regulated genes is shown using ShinyGO [28]. C: Congruent genes with the highest levels of protein-protein interaction (determined via STRING [29]) are plotted in Cytoscape [30]. Interactions are highlighted by lines. Common upregulated genes are shown in red and AD and model downregulated genes are show in blue. Increased size of font for gene symbol reflects higher number of connections between genes. Common genes of interest include: GFAP, TLR4, PTPRC, ITGAM, and TYROBP. Note that the majority of highly connected genes between the two models are upregulated (in red). D: A Venn diagram [48] highlights overlapping genes in the AD portrait and the top two models.

The top two models, APP/PS1 and GFAP mutation, were compared with one another and as shown in Fig 3A display high congruence for both up and down regulated genes. Given that the APP/PS1 mutations lead to increased expression of GFAP and GFAP elevation is also a hallmark of the GFAP mutation, it is possible that many of the shared alterations of gene expression in the two models are related to GFAP expression changes and the astrocyte response. For the top two models, 610 upregulated genes matched up genes (out of the top 1000 up for both) and 388 downregulated genes matched. Only 16 genes were in the opposite direction. An RRHO heatmap of the relation of the top two models is shown in Fig 3A. The 998 congruent genes showed enrichment in a wide range of categories including leukocyte activation, exocytosis, cell adhesion, and kinase binding (Fig 3B). STRING analysis revealed a subset of highly connected genes (Fig 3C) that included PTPRC, ITGAM, TLR4, TLR2, and GFAP. A Venn diagram map of the approximate overlap of the AD portrait and the two models is shown in Fig 3D. Genes altered only in APP/PS1, but not GFAP mutation models, relative to humans with AD, could reflect APP/PSEN1 actions upstream of astrocyte activation and some of the highly interactive genes only found in the APP/PS1 model were: SYN1, GRIA1, GRIA2, BDNF, GAD2, HPCA, PPP2CA, CACNG3, and NRXN3 (Fig 3C). When examining common genes up and down in both models within the canonical AD pathway from KEGG [47], there were 24 locations of exact matches and these were distributed across the pathway (S1 Fig). Eight sites in the KEGG pathway were only found in the GFAP mice when compared with the AD portrait, while three sites were only found in the APP/PS1 mice when compared with the AD portrait.

Other AD-related models had a wide range of matching with AD. The 5XFAD model that has five mutations in APP and PSEN1 on a mixed background ranked 6^th^ overall, but on other backgrounds the score was lower. Scoring highly were mutations for MAPT and APP. The CK-p25 models scored well as did the APOE3 and 4 mutations when they were combined with traumatic brain injury (TBI).

Additional top models included mice with paternal deletion of SNORD116 and IPW exons (PWScrm+/-), a model for Prader-Willi syndrome, SOD1G93A strain mice, treatment with TDP-43 antisense, injection with sporadic Creutzfeldt-Jakob disease tissue, bulbectomized rats, knockout (KO) mice for the ionotropic glutamate receptor subunit (GRID2), KO mice for the zinc finger protein INSM1, KO mice for the transcription factor XBP1, KO mice for the cystatin gene, CSTB, and mice receiving the neurotoxin, MPTP (Table 1). A Flinders sensitive line model ranked highly, but this should be interpreted cautiously as each of four other entries for Flinders were among the most discordant with AD. Aging in mice was the 66^th^ best model.

**Fig 4:**
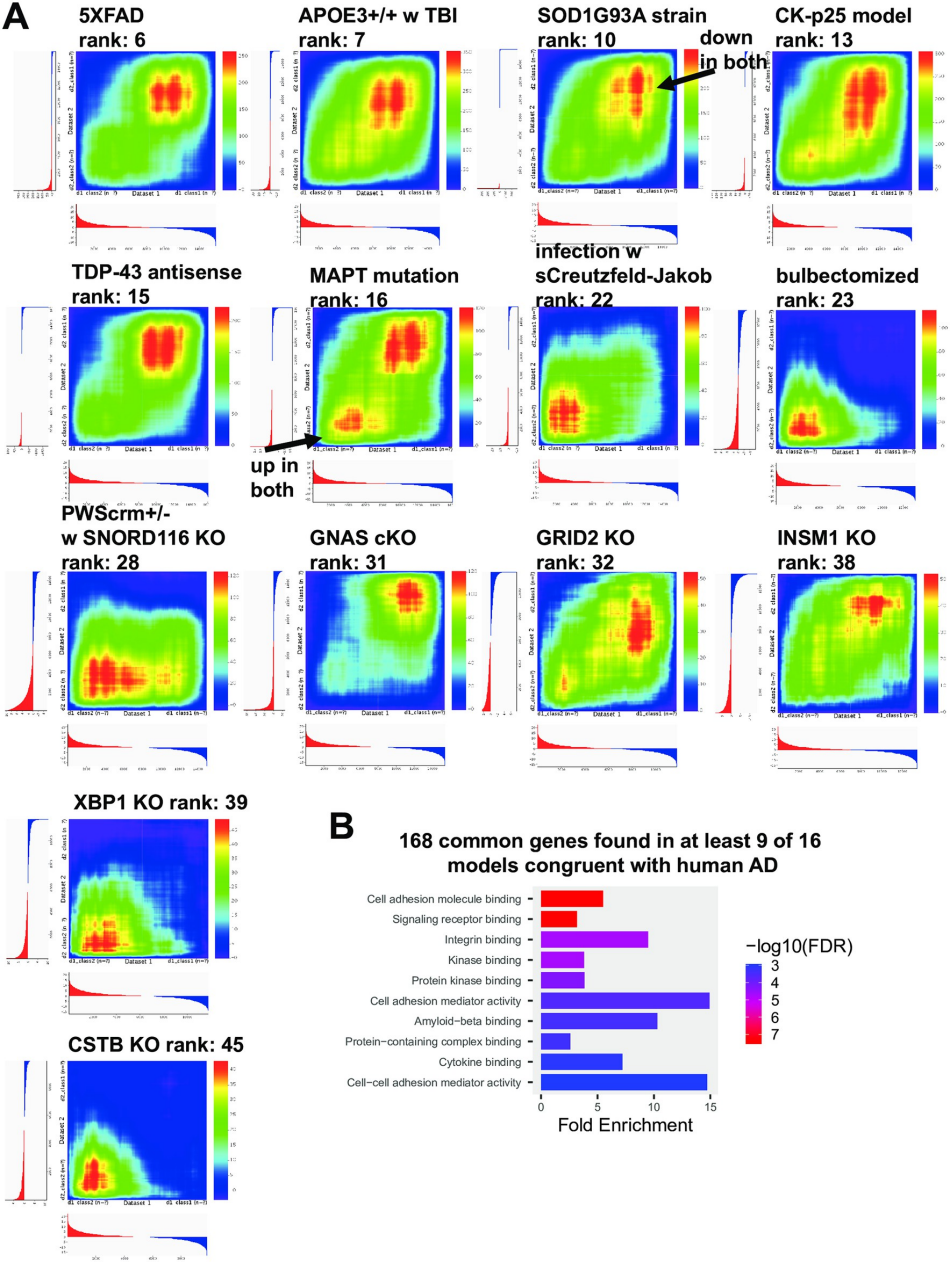
Heat maps of congruent models with AD and common genes among models. RRHO heatmaps [26] (human AD is X-axis) of additional congruent models with AD include: 5XFAD, APOE3 mutation with TBI; SOD1G93A strain, CK-p25 model, TDP-43 antisense treatment, MAPT mutation, infection with sporadic Creutzfeld-Jakob tissue, bulbectomized, PWScrm+/-, GNAS conditional KO, GRID2 KO, INSM1 KO, XBP1 KO, and CSTB KO (A). For the models shown and APP/PS1 and GFAP mutations, common genes with AD from at least 9 of the 16 models were identified (see S2 File) and ShinyGO [28] enrichment analysis was performed (B).

RRHO heatmaps for 14 models (from the top 40) compared with human AD are shown in Fig 4A. From these models as well as the top two models (APP/PS1 and GFAP mutation), common up (N = 126) and down regulated genes (N = 42) with AD found in at least 9 models were identified and the most connected of these genes included TLR4, PTPRC, CD44, STAT3, TLR2, ITGB1, CXCR4, ITGB2, SPP1, FCGR2A, ANXA2, CSF1, and GFAP (Fig 4B). Enrichment analysis of the common genes is shown in Fig 4B and categories included cell adhesion molecule binding and amyloid-beta binding. For a subset of the top models, the specific genes that match AD in the up and the down direction are provided as is a listing of common genes (S2 File).

**Fig 5:**
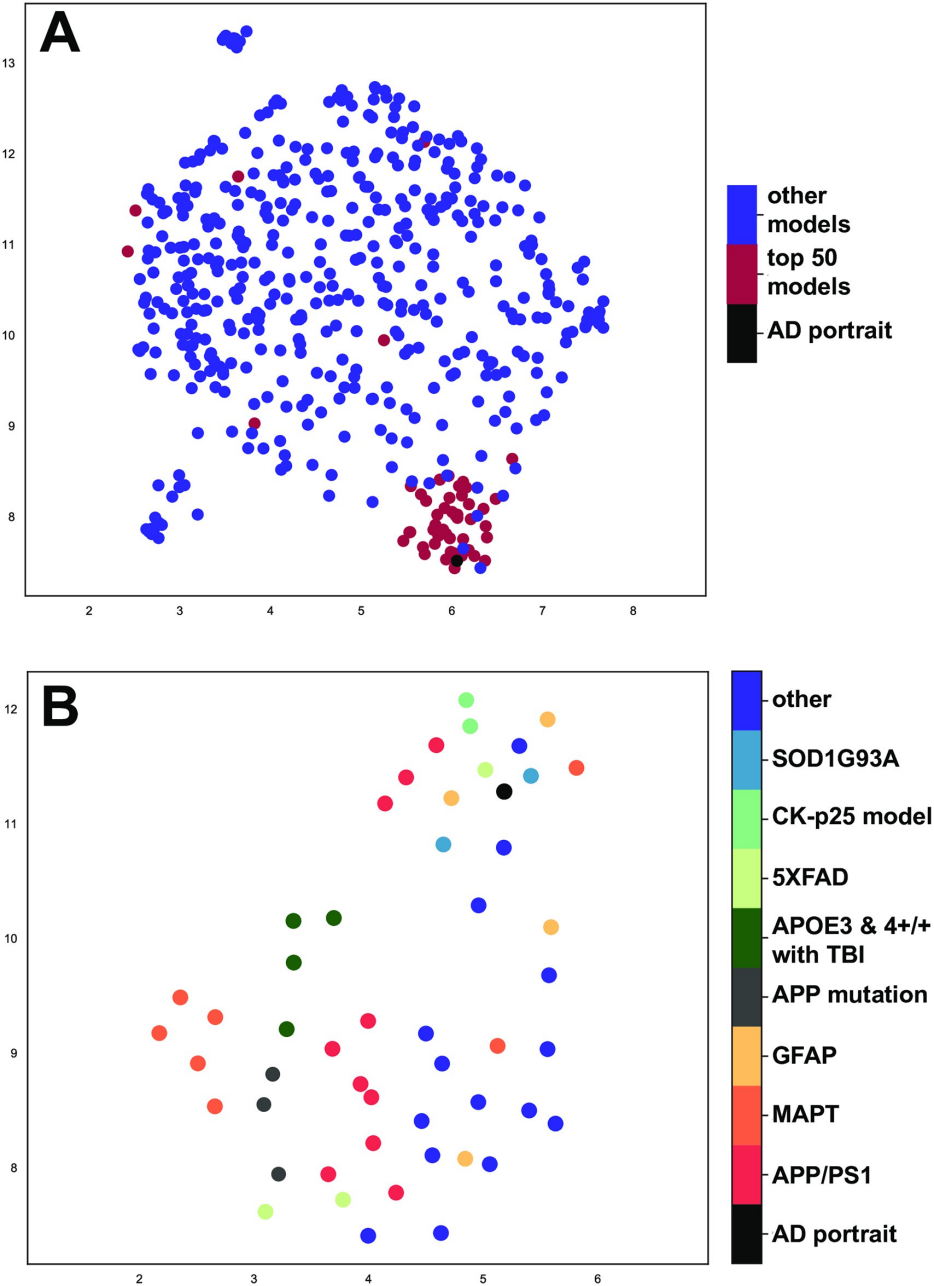
UMAP plotting of models with human AD. UMAP [27] was used to plot (A) all models and (B) just the top 50 models with the AD portrait using 5862 differentially expressed genes. A shorter distance (spatial proximity) of a model with human AD reflects a better match. The X- and Y-axes reflect arbitrary embedding dimensions for A and B. In (A), the top 50 models closely align with the AD portrait. AD portrait circle size was increased 10% and replotted in top surface to emphasize location. In (B), APP/PS1 (orange) and GFAP models (yellow-orange) were congruent with (close spatially to) the human AD (black) as were the 5XFAD (light green), SOD1G93A strain (blue), and the CK-p25 (blue/green) model.

UMAP was used as an additional approach to gain insights into how well models match human AD. In UMAP, closeness spatially represents similarity and when all models are plotted, the top 50 are most closely located next to the AD portrait (Fig 5A). When only the top 50 are plotted, the APP/PS1 and GFAP entries are closely aligned with the AD portrait as are the SOD1G93A strain, 5XFAD, and CK-p25 AD model with the AD portrait (Fig 5B).

### Evaluation of models congruent with additional representations of AD

When human AD was represented by three other meta-analysis studies instead of the AD portrait the results were similar to that with the AD portrait (S3 File). For two studies [16, 17], APP/PS1 and GFAP mutations were the top two models and in the latter case GFAP ranked first. For the third study when using their random effects output [18], APP/PS1 and GFAP were the first and third models. However, when using their fixed effects output the top two models were for APOE3+/+ and APOE4+/+ paired with TBI and APP/PS1 and GFAP were the third and fifth ranked models, respectively (S3 File). When the AD portrait was created using MetaVolcano or the AD portrait was updated with new datasets the top two models were APP/PS1 and GFAP (S3 File).

Male and female AD portraits matched the models in a similar manner as did the AD portrait (S3 File). For three of the four independent studies that had the highest numbers of individuals, namely GSE33000, Mayo clinic, and Mount Sinai Brain Bank (MSBB) studies, males and females also had similar matching to models. For the Religious Order Study and the Memory and Aging Project (ROSMAP) study, females matched models similarly as the other three studies, but males did not (S3 File).

Twenty-eight human AD datasets were compared with each of the animal models (S3 File). Brain region information is provided for humans and animals so direct region to region comparisons can be made. The top models congruent with the AD portrait were found to be congruent with a majority of the individual datasets. The brain region examined was not a major factor in terms of congruence. For example, when examining the ROSMAP dataset for females with AD from prefrontal cortex, the top match was to the APP/PS1 model from CA1 hippocampus (S3 File). Any given human dataset and model dataset from the same or different region can be explored.

When examining subtypes of human AD [19], in our analysis, the C1 and C2 subtypes matched with models in a manner highly similar to the AD portrait with the top matches being the GFAP mutation and the second best match being the APP/PS1 mutation mice. For the A and B1 subtype, an almost opposite pattern of matching models was found relative to the other subtypes with the ATG7 conditional KO as the top match. For the B2 subtype, a large number of Huntington’s disease models were the best matches. HDAC1 mice had a strong match with A, B1, and B2 which is consistent with the finding from Neff et al. [19]. A full list of matching of the models with each human subtype is provided in S3 File.

### Evaluation of models discordant with AD

As a final step we also examined models that had the highest score for discordance (or opposite patterns) with the AD portrait with the idea that these alterations could be neuroprotective and reverse patterns of AD. For each AD dataset examined with the models (S1 and S3 Files), one can examine the discordant models by looking for the top negative scores. Among these models were a KO of TYROBP and a conditional KO of ATG7.

## Discussion

In this study we present new gene expression data from GFAP mutation mice and evaluate congruence of animal models with human AD based on large-scale gene expression patterns in the CNS. The best matches to AD were from the APP/PS1 transgenic mouse model [10]. The mutations are from the APP and PSEN1 genes in humans that are associated with early onset AD [49, 50]. Given that the AD portrait is derived from multiple studies in which most of the individuals had no known mutations [15], the findings of high APP/PS1 congruence with AD suggests the model is relevant for studying AD regardless of its underlying causes in humans. For the top APP/PS1 model, a high number of matches were found with human AD in the same direction (over 500 genes) with very few in the opposite direction (Fig 1). The congruent genes were enriched in categories related to AD and highly connected genes were identified. Although ITPKB (inositol trisphosphate 3-kinase B) was not identified as having a high number of connections in STRING, ITPKB was previously found to be upregulated and the top dysregulated gene in the AD portrait [15]. ITPKB was also upregulated in the APP/PS1 model and is of interest as perturbations of this alone could lead to widespread actions [51–54].

For the GFAP mutation model of AxD, over 500 genes matched AD in the same direction with very few in the opposite direction. An RRHO heatmap of the relation of the top GFAP model with the AD portrait (Fig 2A) highlights the extent of congruence across all genes. The congruent genes were enriched in a wide range of categories that relate to AD (Fig 2B; S2 File) which indicates how this single mutation produces a robust AD-like phenotype at the gene expression level. Congruent genes that showed the highest levels of connections (per STRING) included GFAP, EGFR, TLR4, SNAP25, PTPRC, SLC6A1, CD44, and SNCA (Fig 2C) and it is possible these genes have a strong contribution to the GFAP mutation brain profile that mirrors AD.

GFAP is commonly considered a marker for astrogliosis, and other transcripts associated with gliosis [55–57], including CD44, VIM, OSMR, CP, S1PR3, HSPB1, are elevated in the human AD portrait and matching mouse models, suggesting reactive astrocytes could contribute to the profile. Recent work demonstrating a divergence in the astrocyte response to different neurological disorders [58] further highlights the significance of the matching between AD and mouse models of AxD. Transcripts associated with microglia or myeloid lineage cells are also elevated, as well as innate immune response transcripts, such as toll receptors and interferon responsive genes, many of which could be expressed by either astrocytes or microglia.

Before GFAP mutations were discovered to cause AxD, a common pathogenesis with AD was suggested based on the oxidative injury response, including advanced glycation end products and lipid peroxidation adducts associated with both Rosenthal fibers, the hallmark GFAP aggregates in AxD, and neurofibrillary tangles in AD [59]. Advanced glycation end products/RAGE pathway transcripts are enriched among congruent upregulated genes (S2 File). Astrocytes in AD also display features of cellular senescence [60]. We have recently demonstrated a senescence phenotype in AxD astrocytes in multiple models and patients with the disease [61], and transcripts matching between AxD models and the AD portrait also overlap with those from oxidative stress-induced senescent human astrocytes in vitro [62]. It is noteworthy that not only are young adult AxD mouse models a high match with the AD portrait, but also that astrocytes from these AxD mice during early postnatal development show extensive matching with a neurodegenerative disease of aging. The epsilon isoform of human GFAP (also known as delta) has been shown to interact with presenilin proteins [63]. Whether presenilin binds to mouse GFAP-epsilon, where the tail sequence of the protein is not conserved, has not been determined, and the implications are not clear.

Links between AxD and AD have previously been noted based on common features of oxidative stress [59], but comparisons of clinical symptoms are more difficult to interpret. Progressive dementia is the dominant characteristic of AD [64], and cognitive dysfunction is present in the majority of cases of AxD [65]. However, AxD almost always involves abnormalities of white matter (hence its classification among the leukodystrophies) and significant pathology in the brainstem and spinal cord [66], and its clinical symptomatology is understandably more diverse than that of AD. In AxD, ages of onset range from pre-natal through the 8^th^ decade of life and there are at least two distinct patterns of neuropathology. One recent system for classification described three types, including cerebral, bulbospinal, and intermediate [67]. The cerebral form is typically early onset, and causes seizures, psychomotor developmental delays and regression, dysarthria, dysphagia, and hyperreflexia, whereas the bulbospinal form is later onset, displays less cognitive dysfunction (sometimes with dementia [68]), but with consistent brainstem symptoms including dysphagia, dysphonia, palatal myoclonus, and autonomic symptoms such as constipation and abnormal regulation of body temperature.

The top two models, APP/PS1 and GFAP mutation mice, were highly congruent with one another (Fig 3) and given that both lead to elevated GFAP, it is possible that many of the shared patterns are downstream of increased GFAP expression changes. The congruent genes also showed enrichment in a wide range of categories that relate to AD, and in addition to GFAP, highly connected genes include PTPRC, ITGAM, TLR4, and TLR2. As mentioned above, AxD has similarities with AD, including astrocyte dysfunction [22], and it is possible that there are more underlying connections between the two diseases than previously thought. A Venn diagram map of the approximate overlap of the AD portrait and the two models is shown in Fig 3D and this highlights the high levels of overlap of the three datasets, but also how a theoretical combination of GFAP overexpression and APP/PS1 mutations could provide an even stronger model of AD. The UMAP findings (Fig 5) reflect those of the hypergeometric testing and highlight the congruence of the APP/PS1 and GFAP models with AD. An additional value of the GFAP model is that it may allow for identification of AD-related processes that are upstream of astrocyte or microglia overactivation. Some of the highly connected genes found only in the APP/PS1 mice, but not the GFAP mice, could reflect early effects of the APP and PSEN1 mutations and these genes included BDNF, two glutamate AMPA receptor subunits, GRIA1 and GRIA2, the synaptic vesicle protein, SYN1, the calcium binding protein, HPCA, the GABA producing enzyme, GAD2, the phosphatase, PPP2CA, and the cell adhesion molecule, NRXN3. However, it was also noteworthy that when comparing the models with one another for genes found up or down in both, there was a high matching with the AD KEGG pathway (24 identical sites) that were distributed in various parts of the AD pathway (S1 Fig). The sequence of events that leads to the high similarities of the APP/PS1 and GFAP mutation models remains to be clarified.

Additional datasets from the top 20 models matching the AD portrait included the SOD1G93A mouse model of ALS [34] as well as antisense oligonucleotides to knock down TDP-43 [37], an RNA/DNA binding protein also implicated in ALS. Inclusions containing TDP-43 are prominent pathological features in both ALS and frontotemporal dementia, but can also be found in AxD [69], AD, and dementia with Lewy bodies [70], suggesting a commonality among neurodegenerative proteinopathies [71]. A model of sporadic Creutzfeldt-Jakob disease [38] had high congruence with AD, potentially highlighting the relationship between prion disease, protein misfolding, and amyloid that suggests overlaps of prion disease and AD. The match of bulbectomized rats [39] (Fig 4) is interesting as decreases in sense of smell are associated with AD, including in the early stages [72]. The finding is consistent with the idea that decreased olfactory sensing could contribute to AD emergence. However, if decreased smell is a side effect of AD, then matching with the model could mostly reflect how gene expression is altered when olfactory inputs decrease. Mice with paternal deletion of SNORD116 and IPW exons (PWScrm+/-) that are associated with Prader-Willi syndrome [41] matched AD, and this is consistent with the finding of increased brain aging in adults with Prader-Willi syndrome [73]. KO mice for the ionotropic glutamate receptor subunit (GRID2) [43] were also congruent with AD at high levels (Fig 4) and this suggests possible roles for glutamate and the NMDA receptor in the etiology of AD [74]. KO mice for the zinc finger protein, INSM1 [44], matched AD and this could relate to the role this gene plays in nervous system development. The high matching of AD with the KO mice for the transcription factor XBP1 [45] could relate to the close interactions of XBP1 and BDNF or the role of XBP1 in oxidative stress [75]. Mice receiving the neurotoxin, MPTP, as a means of producing a Parkinson’s disease model [46] matched AD and this could reflect common patterns found across neurodegenerative diseases. The loss of the cystatin gene, CSTB, is a model for progressive myoclonus epilepsy which is an inherited neurodegenerative disease [76] and its congruence with AD also may reflect common patterns found in neurodegenerative disorders. A conditional KO of the tumor suppressor gene GNAS [42] may match AD due to influences on cell proliferation. A Flinders sensitive line model ranked 8^th^ [33], but this should be interpreted cautiously as each of four other entries for Flinders were among the most discordant with AD.

RRHO heatmaps for 14 models (from the top 40) compared with human AD (Fig 4) highlight how some models match well both up and down AD genes, while others mostly match up or down genes. From these models as well as the APP/PS1 and GFAP models, common up and down with AD genes found in at least 9 model datasets were identified that included GFAP, LAMP2, AQP4, CD44, TLR2, and TLR4 (S2 File). TRL2 and TRL4 are both of high interest as they are members of the toll-like receptor family and are involved in pathogen recognition and innate immunity [77, 78]. Further, both TRL2 and TRL4 have been specifically linked to AD and are possible targets for therapeutics [79–82]. That multiple models show elevation of both TRL2 and TRL4 along with AD-like expression profiles suggests a possible causal link of these genes in the emergence of AD. The elevation of GFAP across multiple models matching AD is consistent with an important contributory role of GFAP in AD. Both GFAP and CD44 are markers of gliosis, and AQP4 is expressed by astrocytes.

The 5XFAD model that has five mutations in APP and PSEN1 scored highly (ranked 6^th^) when on multiple mixed (BxD) genetic backgrounds [31], but matched at a lower level (rank = 41) when on a C57BL/6J background [83]. This finding is consistent with work showing that the 5XFAD best reflects the AD phenotype when on a mixed background and that the C57 strain may be resilient to the mutations [31]. For additional AD-related models, scoring highly were mutations for MAPT and APP and this is consistent with roles for both genes in AD as well as prior work linking various MAPT and APP models to AD [84, 85]. The CK-p25 models scored well which matches the earlier report of this model’s congruence with human AD [35]. The finding that the combination of TBI with the APOE3 and 4 late onset AD mutations [32] matched AD reflects how environmental perturbations can adversely affect certain genotypes over others. In recent work, multiple AD-related models were congruent with a human depression portrait [25], including APPsa knock-in, APP knockout, APP/PS1 mutation, and APLP2 knockout and this is consistent with the finding of overlaps between AD and depression portraits [15]. Why and how the different AD-related models match AD and depression at different levels still needs to be determined.

Overall, model ranking was similar for the AD portrait and three other meta-analysis studies by others (S3 File). The top models were also similar across individual datasets, including those from specific CNS regions. The matching of models with male and female AD portraits was similar and this was also the case for three of the four independent studies that had the highest numbers of individuals per study (S3 File). For the ROSMAP study [86–88], females matched models as found in other studies, but males did not (S3 File). In recent work, a gene expression sex difference in male and female rTg4510 (MAPT mutation) mice was found in terms of aging and matching a human AD coexpression module [18]. In future studies, a comprehensive analysis of sex differences may provide critical new information.

The present work complements a recent study that evaluated over 200 mouse models for AD and that focused on human AD-related weighted correlation network analysis (WGCNA) co-expression modules from different brain regions [18]. In that study, models with the MAPT, the CDK5-p25, and the 5XFAD mutations matched multiple human co-expression AD modules. Further, models for other diseases, such as ALS and Huntington’s disease were also identified as matching AD modules. One unique aspect of the current study was our use of a standardized differential expression cutoff across models and in the human AD datasets being tested that was independent of a gene’s inclusion in a module. There are values in both approaches whereby our approach first allows identification of matches but then requires downstream analysis of matching (such as enrichment or gene network analysis) while the modules approach allows direct identification of matching with known gene networks in AD. In future studies, it may be useful to combine both approaches to allow for full evaluation of extent of matching of models to human AD, but also to know the extent of matching to key AD co-expression modules.

Our study also complements a second study that examined human subtypes of AD based on gene expression patterns and dysregulated molecular pathways [19]. In their report, amyloid-β related subtypes C1 and C2, which appeared to be driven by inflammatory processes, were the most robust and matched well the APP/PS1 and 5XFAD models [19]. We also found C1 and C2 to be highly congruent with the APP/PS1 and 5XFAD models (S3 File). The finding of a high number of Huntington’s disease models that matched the B2 group, corresponding to tau-related neurodegeneration, is consistent with earlier work finding similarities between AD and Huntington’s disease [14]. The A and B1 subtype matching to models was almost opposite to the other subtypes. The top A and B1 match was ATG7 conditional KO in motor neurons and as discussed below the ATG7 model has an overall reversed expression pattern relative to most representations of AD. Further exploration of how AD subtypes or AD pathways match models is a promising direction of study.

This work also allowed us to identify possible genes that were neuroprotective and produced expression patterns opposite to that of AD. Interestingly, the ATG7 conditional KO in motor neurons had already been found in the original study to extend the lifespan and reduced glial inflammation when placed in the SOD1G93A mouse model of ALS [34]. Similarly, the loss of TYROBP reversed AD gene expression patterns, consistent with the original finding that this manipulation is neuroprotective, including amelioration of learning deficits, when placed in the APP/PS1 line [89]. TYROBP was also found to be consistently upregulated in a number of the best models (Fig 4B). How reduced ATG7 and TYROBP expression act to reverse some patterns of AD could be a promising area of research.

This study also includes multiple technical limitations. In some cases, two genotypes, such as KO and wild-type, were both given vehicle injections and it is possible that the vehicle itself or process of injection led to alterations in gene expression patterns. Although a few models were tested at multiple ages, most were not, so how expression changes with aging and how that may relate to AD is not known. Not all model study data are available and it is possible relevant datasets were missed. Finally, not all genes in humans have direct homology in animal models, so some aspects of AD cannot be evaluated using a model approach.

## Conclusions

In this study, we identify multiple animal models with strong matching to AD at the large-scale gene expression level, including the top two, APP/PS1 and GFAP models. That these models also match one another at a high level is interesting given that the genes are in different pathways. However, both models lead to elevated levels of GFAP and one possibility is that many of the consequences of APP/PS1 mutations are downstream of astrocyte activation. The identification of common genes dysregulated in most of the top models highlights possible drivers of the AD expression profiles. Finally, our finding that some gene manipulations reverse AD expression patterns, as with TYROBP and ATG7, adds to work indicating these as potential targets for treatments. The study contributes to on-going work by others evaluating animal models’ expression patterns with AD to help understand the pathways and causes of AD.

## Materials and methods

### Animal model datasets

Five hundred and five animal models were evaluated, including new data from GFAP mutation mice described below. Three hundred and fifty two animal datasets included ones we previously evaluated for congruence with human depression [25] as well as newly entered models that are relevant to AD as well as other neurological disorders, such as Parkinson’s disease. We used the Crowd feature in Enrichr [90] along with genes from the human AD portrait [15] to help identify potential AD models. Most datasets were obtained from GEO RNA-seq Experiments Interactive Navigator (GREIN) [91] or the Gene Expression Omnibus (GEO) [92]. One entry for 5XFAD came from a supplementary file within [31] that performed differential expression between multiple BxD strains with mutations relative to controls. One hundred and fifty two additional models from a recent study also analyzing relevance of mouse models to AD [18] and we used those that were available via Synapse.org (see links and acknowledgments below). In cases where datasets from Synapse were duplicates of our original datasets, we used our datasets and these included: GSE100070, GSE42912, GSE54795, GSE63943, GSE75431, GSE76567, GSE77681, and GSE87202. For naming, both lists began each model name with an “m”, so to distinguish the two and to allow for reference to prior publications, we keep our naming (e.g., m1, m2, etc) and capitalize the second list and end it with an A (e.g., M12A, M13A, etc). For the second list, the exact naming of each model from the original study is provided under “source; notes” column of the relevant Supplementary Files. In some cases, we shortened the description under the “genotype or treatment” column for list 2, but the listing under “source;notes” column should be considered the most accurate for list 2. For the 505 combined models, datasets were used that assessed expression in nervous system related tissue (e.g., CNS, olfactory bulb, retina, multiple brain regions). When possible, males and females were analyzed separately, but not all studies provided information on sex. The age of cohorts is also provided under the “source;notes” column. However, in some cases age is not provided in the original study. Gene symbols for each dataset were updated to HUGO [93] so comparisons to humans could be made. Further, for each dataset p-values were -log10 transformed so that a larger number indicates greater significance. This number was multiplied by the sign of direction change to allow for comparison of up versus down regulated genes. Although microarray and RNA sequencing data require different differential expression approaches every attempt was made to use consistent standards across studies.

### Mouse tissue collection and RNA sequencing

All animal studies were approved by the College of Letters and Sciences and Vice Chancellor Office for Research and Graduate Education Animal Care and Use Committee at the University of Wisconsin-Madison. All experiments are in compliance with relevant guidelines and regulations as well as the current edition of the Guide for the Care and Use of Laboratory Animals [94].

Adult male wild-type Gfap+/+ and Gfap+/R236H littermates in the FVB/N-Taconic genetic background [23] were sacrificed at day postnatal 56 or 57 by CO2 asphyxiation (N = 4 per group), and brains removed and rapidly microdissected on ice. Hippocampus and corpus callosum were frozen on dry ice and stored at -80°C before homogenization in Trizol for RNA extraction according to the manufacturer’s protocol (Invitrogen, ThermoFisher Scientific). RNA was treated with TURBO DNase (TURBO DNA-free Kit, Ambion, ThermoFisher Scientific) before measuring RNA quality with an Agilent 2100 Bioanalyzer, and RNA integrity numbers (RIN) were between 8.1 and 9.2. Libraries were prepared from 0.5 (corpus callosum) or 1 ug (hippocampus) total RNA for sequencing with TruSeq Stranded Total RNA Sample Preparation kit with ribosomal RNA reduction (Illumina). Libraries were quantified (Qubit) and assayed with the Bioanalyzer to confirm integrity before sequencing with an Illumina NovaSeq 6000 (2x150bp, 70M reads per sample, 1 lane of S4 flowcell). Base calling was performed using Bcl2fastq, read trimming with Skewer [95], read alignment with STAR [96], expression estimation with RSEM [97], and differential expression estimation with EdgeR [98]. Results were deposited in the Gene Expression Omnibus repository under accession number GSE197044.

To assess astrocyte specific gene expression during postnatal development in GFAP mutation mice, we performed TRAPseq (translating ribosome affinity purification) using detailed methods previously described by Heiman et al. [99]. Male JD130 mice (C57BL/6J) expressing RPL10a fused to EGFP under the control of the Aldh1l1 promoter [100] (kind gift from Nathaniel Heintz) were crossed with female Gfap+/R236H mice (FVB/N-Tac) and male FVBB6F1 pups sacrificed for tissues at postnatal day14. Hippocampus and corpus callosum were collected and stored frozen as described above. Translating ribosomes were isolated using antibodies against EGFP (Htz-GFP-19F7 and Htz-GFP-19C8, Memorial Sloan-Kettering Monoclonal Antibody Facility, bioreactor supernatant purity) to immunoprecipitate the EGFP-L10a fusion protein, and the associated RNA was purified (Agilent Absolutely RNA Microprep kit, including DNase step) for sequencing and gene expression analysis. Libraries for sequencing were prepared with 100 ng RNA using standard protocols for the TruSeq Stranded mRNA kit with poly-A selection (Illumina). Sequencing was performed (1x100bp) using an Illumina HiSeq2500 at 25M reads per sample with each sample consisting of tissues pooled from 5 mice, N = 3 samples (15 mice) for each genotype. Reads were mapped back to the genome using Bowtie [101], followed by RSEM to estimate gene expression. Results were deposited in the Gene Expression Omnibus repository under accession number GSE198817.

### Human AD datasets and comparison with animal models’ gene expression patterns

AD in the CNS in humans was represented by multiple datasets. From our recent study [15], we used an AD portrait derived from 22 male and female AD datasets across multiple brain regions, an AD portrait made using metaVolcano approaches [102], and a modified AD portrait that blended our original AD portrait with six additional datasets with harmonized differential expression [87] from the Mayo [103], ROSMAP [86, 88], and MSBB studies [104]. We also used three other recent meta-analysis studies of AD [16–18]. Male and female specific portraits of AD from our recent study [15] were each updated with three new male and female datasets from the Mayo, MSBB, and ROSMAP studies [87] (see S3 File for details). Our approach for creating a portrait from multiple datasets is to treat each dataset equally and identify genes that move consistently in one direction (up or down) across studies [15]. Additionally, 28 individual AD datasets that are region specific were used (S3 File). We also used data from a study subdividing AD into subtypes and in this one case the genes were ordered by extent and direction of fold change from control [19].

For quantitative analysis of an animal model dataset with a human AD dataset we used a rank rank hypergeometric approach that evaluated the top 1000 upregulated and top 1000 downregulated genes. Hypergeometric analysis was run in R [105] and the evaluation followed that of the hypergeometric publication [26]: A, up in both datasets; B, up in model, but down in AD; C, down in model, but up in AD; D, down in both datasets. The p-value was -log10 transformed so that a larger number indicates greater significance of overlapping genes and the output of A and D were added together to highlight common directions in the datasets. From that score the scores from B and C (opposite directions in datasets) were subtracted to provide a final score with a higher number indicating greater congruence between the two datasets. The cutoff of 1000 is expected to reflect reliable biological alterations [106] and a specific number allows equal analysis for all datasets. The outputs of the scoring of all the models to the different representations of human AD are provided in S1 and S3 Files.

RRHO [26] was employed to create heat maps between datasets. RRHO reflects data from all genes and highlights genes in the same and opposite directions. We also used UMAP analysis [27] to evaluate congruence between models and human AD. Similar datasets are plotted more closely to one another. We used data from the top 5862 genes from the human AD portrait that were also found in the majority of the models using the correlation function and plotting either all models or just the top 50 with the AD portrait. UMAP was run in Python 4.01 [107] using Anaconda (Spyder) [108].

### Analysis of congruent genes

For enrichment of genes that were either up-up or down-down in two datasets, ToppCluster [109], ShinyGo 0.77 [28], or STRING [29] were employed. Genes were also entered into STRING and highly interacting proteins (minimum interaction score of 0.40) were identified and exported to Cytoscape [30] for visualization using yFiles Radial Layout [110]. The AD pathway from KEGG [47] was used to identify where common genes from the APP/PS1 and GFAP mutation mice that matched the AD portrait were located within this spatial mapping of AD.

## Supporting information

S1 Fig Common APP/PS1 and GFAP genes in the AD KEGG pathway. Using analysis of the AD pathway within KEGG [47], twenty four common sites (in red bars) are found for where the same gene is perturbed in the same direction in the two genotypes and matches the AD KEGG pathway. Permission to use the copyrighted image was provided by KEGG [47]. Additional sites in the KEGG pathway are matched by genes only found in the same direction for APP/PS1 and the AD portrait (green star) or GFAP and the AD portrait (blue star). The common sites of action of GFAP and APP/PS1 are distributed across multiple sites in the AD pathway highlighting that while different mutations of different genes start the dysregulation, there is strong convergence of action.
(PDF)

S1 File Scoring and ranking of animal models with AD portrait; GFAP mutation differential gene expression results. Scoring and ranking of over 500 models are compared with the AD portrait based on a hypergeometric test. Highest positive score represents the highest model match. Genes in the same direction are rewarded in scoring while genes in the opposite direction are penalized. Analysis also allows for identification of models that are most discordant with AD where patterns of genes are in the opposite direction. Differential gene expression of hippocampus and corpus collosum comparing GFAP mutation mouse model with control.
(XLSX)

S2 File Enrichment analysis of congruent genes in top models and AD portrait. ToppCluster analysis [109] of genes from top ranking models that match human AD in the same direction. Common genes in multiple top models for AD are identified.
(XLSX)

S3 File Scoring and ranking of animal models with multiple representations of human AD. Human AD is represented by over 40 datasets, including meta-analysis from other studies, portraits for female AD and male AD, and individual datasets that are region and sex specific. Each of these is compared with the over 500 animal models and a ranking of the top models is provided based on the extent of similar gene expression patterns in the same direction.
(XLSX)
