# Supplementary figures and images for: Large-scale gene expression changes in APP/PSEN1 and GFAP mutation models exhibit high congruence with Alzheimer’s disease
Source: PLoS One. 2024 Jan 18;19(1):e0291995. doi: 10.1371/journal.pone.0291995 (PMC10796008; doi:10.1371/journal.pone.0291995)

# ALZHEIMER DISEASE

■ = both models

★ = GFAP model only  
and AD portrait

★ = APP/PS1 model only  
and AD portrait

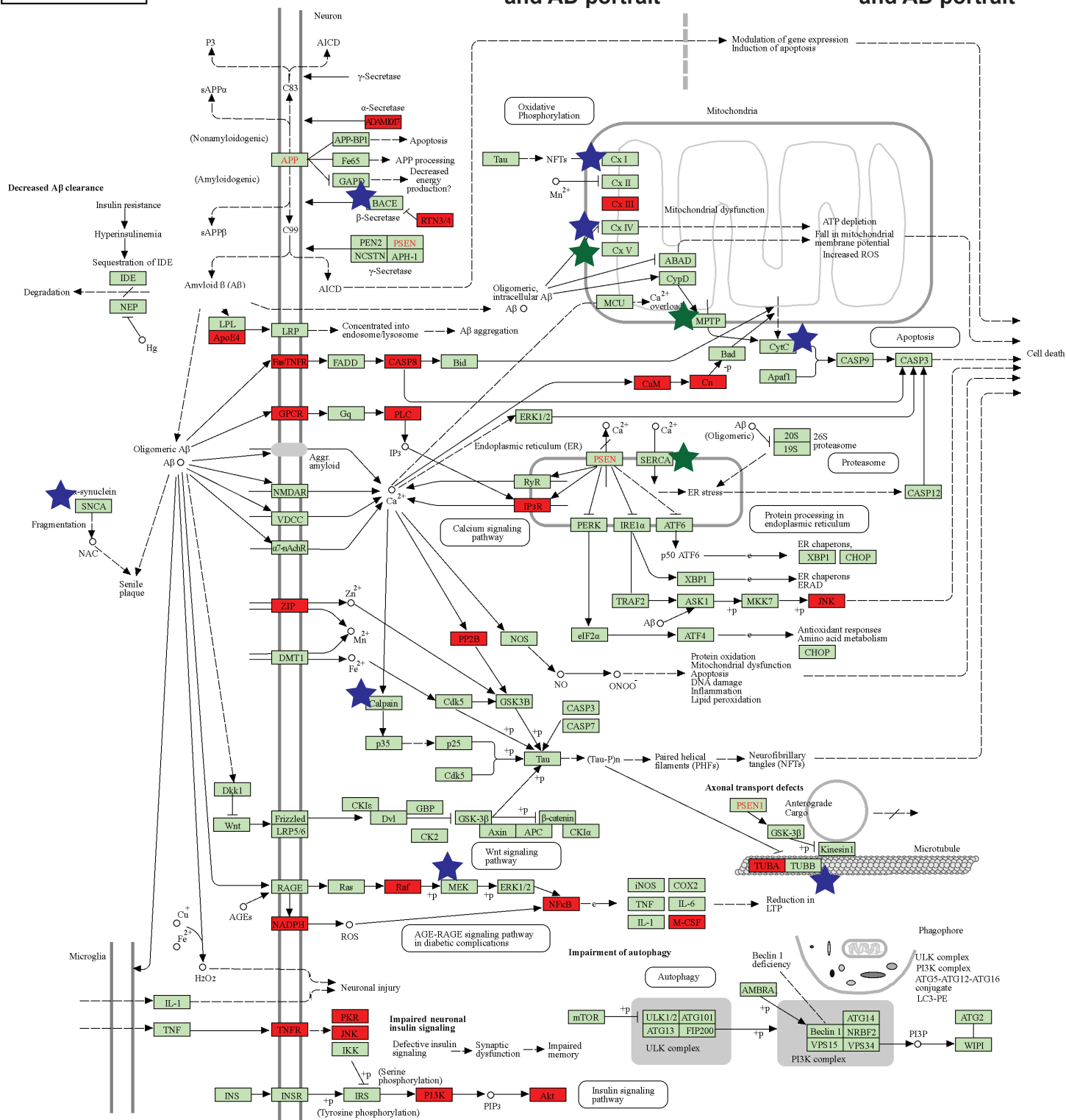

Supplement: S1 Fig — Using analysis of the AD pathway within KEGG [47], twenty four common sites (in red bars) are found for where the same gene is perturbed in the same direction in the two genotypes and matches the AD KEGG pathway. Permission to use the copyrighted image was provided by KEGG [47]. Additional sites in the KEGG pathway are matched by genes only found in the same direction for APP/PS1 and the AD portrait (green star) or GFAP and the AD portrait (blue star). The common sites of action of GFAP and APP/PS1 are distributed across multiple sites in the AD pathway highlighting that while different mutations of different genes start the dysregulation, there is strong convergence of action. (PDF) [file pone.0291995.s001.pdf]
